# Distinct HR expression patterns significantly affect the clinical behavior of metastatic HER2+ breast cancer and degree of benefit from novel anti‐HER2 agents in the real world setting

**DOI:** 10.1002/ijc.32583

**Published:** 2019-08-07

**Authors:** Laura Pizzuti, Eriseld Krasniqi, Giacomo Barchiesi, Marina Della Giulia, Fiorentino Izzo, Giuseppe Sanguineti, Paolo Marchetti, Marco Mazzotta, Raffaele Giusti, Andrea Botticelli, Teresa Gamucci, Clara Natoli, Antonino Grassadonia, Nicola Tinari, Laura Iezzi, Silverio Tomao, Federica Tomao, Giuseppe Tonini, Daniele Santini, Antonio Astone, Andrea Michelotti, Claudia De Angelis, Lucia Mentuccia, Angela Vaccaro, Emanuela Magnolfi, Alain Gelibter, Valentina Magri, Enrico Cortesi, Loretta D'Onofrio, Alessandra Cassano, Ernesto Rossi, Marina Cazzaniga, Luca Moscetti, Claudia Omarini, Federico Piacentini, Maria A. Fabbri, Angelo F. Scinto, Domenico Corsi, Luisa Carbognin, Emilio Bria, Nicla La Verde, Riccardo Samaritani, Carlo Garufi, Sandro Barni, Rosanna Mirabelli, Roberta Sarmiento, Enzo M. Veltri, Giuliana D'Auria, Ida Paris, Francesco Giotta, Vito Lorusso, Franca Cardillo, Elisabetta Landucci, Maria Mauri, Corrado Ficorella, Mario Roselli, Vincenzo Adamo, Giuseppina R.R. Ricciardi, Antonio Russo, Rossana Berardi, Mirco Pistelli, Elena Fiorio, Katia Cannita, Valentina Sini, Nicola D'Ostilio, Jennifer Foglietta, Filippo Greco, Claudio Zamagni, Ornella Garrone, Barbara Di Cocco, Editta Baldini, Lorenzo Livi, Isacco Desideri, Icro Meattini, Giuseppina Sarobba, Pietro Del Medico, Michele De Tursi, Daniele Generali, Ruggero De Maria, Emanuela Risi, Gennaro Ciliberto, Isabella Sperduti, Alice Villa, Maddalena Barba, Angelo Di Leo, Patrizia Vici

**Affiliations:** ^1^ Division of Medical Oncology 2, IRCCS Regina Elena National Cancer Institute Rome Italy; ^2^ Department of Radiation Oncology IRCCS Regina Elena National Cancer Institute Rome Italy; ^3^ Medical Oncology Unit Azienda Ospedaliera Universitaria Sant'Andrea Rome Italy; ^4^ Medical Oncology Unit B Policlinico Umberto I Rome Italy; ^5^ Medical Oncology Sandro Pertini Hospital Rome Italy; ^6^ Department of Medical, Oral and Biotechnological Sciences Centro Scienze dell'Invecchiamento e Medicina Traslazionale ‐CeSI‐MeT Chieti Italy; ^7^ Department of Radiological, Oncological and Anatomo‐Pathological Sciences ‘Sapienza’ University of Rome, Policlinico Umberto I Rome Italy; ^8^ Department of Gynecology‐Obstetrics and Urology “Sapienza” University of Rome Rome Italy; ^9^ Department of Oncology University Campus Biomedico of Rome Rome Italy; ^10^ Division of Medical Oncology Villa San Pietro Hospital Rome Italy; ^11^ UO Oncologia Medica I, S. Chiara Hospital, Dipartimentodi Oncologia, Dei Trapianti e Delle Nuove Tecnologie Azienda Ospedaliera Universitaria Pisana Pisa Italy; ^12^ Medical Oncology Unit ASL Frosinone Frosinone Italy; ^13^ Department of Medical Oncology Policlinico Universitario “A. Gemelli” Rome Italy; ^14^ Universita Cattolica del Sacro Cuore Roma; ^15^ Research Unit Phase I trials and Oncology Unit, ASST Monza Monza Italy; ^16^ Division of Medical Oncology, Department of Oncology and Hematology University Hospital of Modena Modena Italy; ^17^ Medical Oncology Unit Belcolle Hospital Viterbo Italy; ^18^ Medical Oncology Unit Fatebenefratelli Hospital Rome Italy; ^19^ Gynecology Oncology Unit Catholic University of the Sacred Heart Rome Italy; ^20^ Oncology Unit ASST Fatebenefratelli Sacco Presidio Ospedaliero Fatebenefratelli Milano Italy; ^21^ Medical Oncology Unit “Regina Margherita” Hospital Rome Italy; ^22^ Division of Medical Oncology Pescara Hospital Pescara Italy; ^23^ Department of Oncology Oncology Unit, ASST Bergamo Ovest Treviglio Italy; ^24^ Department of Hematology & Oncology Azienda Ospedaliera Pugliese‐Ciaccio Catanzaro Italy; ^25^ Oncology Unit San Filippo Neri Hospital Rome Italy; ^26^ Oncology Unit S. Maria Goretti Hospital Latina Italy; ^27^ Department of Medical Oncology “Giovanni PaoloII” Institute Bari Italy; ^28^ Oncology Unit Formia Hospital ASL Latina Italy; ^29^ Division of Oncology San Giovanni Hospital Rome Italy; ^30^ Medical Oncology, Department of Biotechnological and Applied Clinical Sciences University of L'Aquila L'Aquila Italy; ^31^ Department of Systems Medicine, Medical Oncology University of Rome “Tor Vergata” Rome Italy; ^32^ Medical Oncology Unit A.O. Papardo & Department Human Pathology University of Messina; ^33^ Department of Surgical, Oncological and Oral Sciences, Section of Medical Oncology University of Palermo Palermo Italy; ^34^ Oncology Clinic Università Politecnica delle Marche, Ospedali Riuniti di Ancona Ancona Italy; ^35^ U.O.C. Oncology, University of Verona Azienda Ospedaliera Universitaria Integrata Verona Italy; ^36^ Medical Oncology St. Salvatore Hospital L'Aquila Italy; ^37^ Oncology Unit ASL Roma 1, Santo Spirito Hospital Rome Italy; ^38^ U.O.C. Lanciano‐Vasto Italy; ^39^ Medical Oncology, P.O. Narni‐Amelia Terni Italy; ^40^ Department of Pathology, Surgery and Oncology “Mater Salutis” Hospital, ULSS21 Verona Italy; ^41^ Medical Oncology Unit, Addarii Institute of Oncology S. Orsola‐Malpighi Hospital Bologna Italy; ^42^ Medical Oncology A.O. Ospedale di Insegnamento S. Crocee Carle Cuneo Italy; ^43^ Department of Oncology Lucca Hospital Lucca Italy; ^44^ Radiation Oncology Unit and Department of Clinical and Experimental Biomedical Sciences “Mario Serio”, Azienda Ospedaliera Universitaria Careggi University of Florence Florence Italy; ^45^ Department of Medical Oncology, ASL Nuoro Nuoro Italy; ^46^ Division of Medical Oncology Reggio Calabria General Hospital Reggio Calabria Italy; ^47^ Breast Cancer Unit & Translational Research Unit, ASST Cremona Cremona Italy; ^48^ Sandro Pitigliani Medical Oncology Department Hospital of Prato Prato Italy; ^49^ Scientific Direction IRCCS Regina Elena National Cancer Institute Rome Italy; ^50^ Bio‐Statistics Unit IRCCS Regina Elena National Cancer Institute Rome Italy

**Keywords:** advanced breast cancer, HER2 positive, pertuzumab, trastuzumab, T‐DM1, real world

## Abstract

We analyzed data from 738 HER2‐positive metastatic breast cancer (mbc) patients treated with pertuzumab‐based regimens and/or T‐DM1 at 45 Italian centers. Outcomes were explored in relation to tumor subtype assessed by immunohistochemistry (IHC). The median progression‐free survival at first‐line (mPFS1) was 12 months. Pertuzumab as first‐line conferred longer mPFS1 compared to other first‐line treatments (16 *vs*. 9 months, *p* = 0.0001), regardless of IHC subtype. Median PFS in second‐line (mPFS2) was 7 months, with no difference by IHC subtype, but it was more favorable with T‐DM1 compared to other agents (7 *vs*. 6 months, *p* = 0.03). There was no PFS2 gain in patients with tumors expressing both hormonal receptors (HRs; *p* = 0.17), while a trend emerged for tumors with one HR (*p* = 0.05). Conversely, PFS2 gain was significant in HRs‐negative tumors (*p* = 0.04). Median overall survival (mOS) was 74 months, with no significant differences by IHC subtypes. Survival rates at 2 and 3 years in patients treated with T‐DM1 in second‐line after pertuzumab were significantly lower compared to pertuzumab‐naïve patients (*p* = 0.01). When analyzed by IHC subtype, the outcome was confirmed if both HRs or no HRs were expressed (*p* = 0.02 and *p* = 0.006, respectively). Our results confirm that HRs expression impacts the clinical behavior and novel treatment‐related outcomes of HER2‐positive tumors when treatment sequences are considered. Moreover, multivariate analysis showed that HRs expression had no effect on PFS and OS. Further studies are warranted to confirm our findings and clarify the interplay between HER2 and estrogen receptor pathways in HER2‐positive (mbc) patients.

## Abstract 2

**What's new?**

About half of breast cancers positive for human epidermal growth factor (HER2) also express hormone receptors but the impact of hormone receptor status on the success of HER2‐directed treatments is not fully explored. Here the authors retrospectively assessed tumor behavior and treatment outcomes in 738 women with HER2+ metastatic breast cancer treated with new generation anti‐HER2 agents. Distinct hormone receptor expression patterns significantly affected the progression free and overall survival, justifying further studies to define optimal treatment regimens and the interplay between hormone receptor and HER2 signaling.

**Abbreviations**
BC: breast cancer
CISH: chromogenic *in situ* hybridization
ER: estrogen receptor
FISH: fluorescence *in situ* hybridization
HER2: human epidermal growth factor receptor 2
HR: hormone receptor
IHC: immunohistochemistry
OS: overall survival
PFS: progression‐free survival
PgR: progesterone receptor
TP: triple‐positive

## Introduction

Amplification or overexpression of the human epidermal growth factor receptor 2 (HER2) is observed in approximately 20% of breast cancers (BCs) and is often associated with an aggressive clinical behavior and poor outcomes.^1^ When overexpressed, the HER2 oncogene is the dominant driver of BC biology, and treatment strategies targeting HER2 have become the standard of care in all the disease settings since 2006.^2^ Currently, four anti‐HER2 agents are licensed in Europe: trastuzumab, lapatinib, pertuzumab and T‐DM1. The use of anti‐HER2 agents combined or not with chemotherapy has markedly improved prognosis in all stages of HER2‐positive BC.^3^, ^4^, ^5^, ^6^, ^7^

About half of HER2‐positive BCs also express hormone receptors (HRs).^8^ The coexistence of both estrogen receptors (ER) and progesterone receptors (PgR) expression and overexpression/amplification of HER2 is the hallmark of the subgroup called “triple positive” (TP) breast cancer. Even though treatment with anti‐HER2 agents has shown benefit independently on HR status, the cross‐talk between the two downstream pathways has an impact on the natural history of the disease and magnitude of treatment‐benefit.

In the metastatic setting, recent reports of outcomes by tumor subtype demonstrated that HR‐positive/HER2‐positive tumors usually exhibit different behaviors and response to trastuzumab‐based therapy, as well as distinct timings and patterns of relapse compared to HR‐negative/HER2‐positive tumors.^9^, ^10^ In the CLEOPATRA trial, the addition of pertuzumab to a first‐line regimen with trastuzumab and docetaxel did not show any advantage in terms of overall survival (OS) in the subgroup of patients with ER and/or PgR‐positive tumors (HR 0.73; 95% CI, 0.50–1.06).^9^ Conversely, in the Emilia and in the TH3RESA trials, patients received a significant PFS and OS benefit from the administration of T‐DM1 regardless of HRs expression [7;11]. Yet, no data on novel HER2‐blocking agents’ outcomes have been specifically reported on tumors overexpressing HER2 and expressing both HRs, that is, TP tumors. So, we conducted a retrospective analysis on HER2‐positive metastatic patients treated with pertuzumab‐based regimens and/or T‐DM1 according to standard clinical practice, and we specifically focused on whether the expression of HRs defines distinct subtypes with different biological behaviors and patterns of response/resistance to novel HER2‐blocking agents in HER2‐positive metastatic BCs.

## Materials and Methods

Our cohort included 738 HER2‐positive metastatic BC patients consecutively recruited and treated with pertuzumab‐based regimens and/or T‐DM1 in any treatment line according to routine practice at 45 Italian Oncologic Centers from December 2003 through November 2017. The observational study was carried out according to a retrospective approach. Written informed consents were obtained from all patients providing data to our analysis. Information on demographics, clinical, histopathological and immunohistochemical (IHC) features, antitumoral therapies and related outcomes were retrieved from the patients’ medical records by specifically trained research assistants. All included patients were treated for a metastatic disease (defined as a BC spread over the mammary gland and the pertinent locoregional lymph nodes, including the supraclavicular ones). Each patient was evaluated during treatment according to the follow‐up strategies of each center. In all cases, clinical evaluation, bone scan, computerized tomography (CT) and/or positron emission tomography (PET) – CT were performed at least every 3 months. Clinical response was evaluated by RECIST criteria Anonymized data were entered into a dedicated database with a SPSS operating interface. Median follow up was calculated starting from diagnosis of metastatic disease to death or date at the last follow up. Endpoints for efficacy outcome included progression‐free survival (PFS) and overall survival (OS). PFS for any specific line of treatment was calculated from the time of treatment start to the time of progression of disease, interruption of treatment for toxicity, death or lost to follow‐up. OS was calculated starting from diagnosis of metastatic disease to death or last follow‐up. For both PFS and OS the median value, respectively median PFS (mPFS) and median OS (mOS), were calculated using the Kaplan–Meier limit product method. A further assessment of OS in different patients’ subsets was performed by calculating the OS rate at 2 and 3 years.

Pathology assessment was performed on surgical specimens of primary tumors by dedicated pathologists at the participating centers as per national standards. Estrogen receptor and PgR status were determined at each center by IHC according to local standards. Positivity was considered at a cutoff of ≥1%. A positive HER2 status required an IHC score of 3+ or positive fluorescence *in situ* hybridization/chromogenic *in situ* hybridization (FISH/CISH). Our study was approved by the institutional ethical committee of the coordinating center (IRCCS Regina Elena National Cancer Institute of Rome) and satellite centers and was conducted in compliance with the Helsinki Declaration.

## Statistical Analysis

Descriptive statistics were used to summarize patient‐ and disease‐relevant characteristics. The associations between variables were tested by Chi‐square or Fisher's exact test, according to the number and size of the groups compared. Survival estimates were computed by Kaplan–Meier product‐limit and compared by log‐rank test. Significance was set at *p* ≤ 0.05. The associations of interest were evaluated in light of the distinction of the overall study cohort into categories defined by molecular subgroups, with the inherent modalities being set based on the results of IHC analysis and according to the criteria fully reported in the prior paragraph (Patients and Methods). The impact of the most relevant variables on OS and PFS1 was tested in COX uni/multivariate models. Significance was set at *p* ≤ 0.05. The following variables were considered: ICH subgroup, age, PS, metastatic disease at diagnosis, number of metastatic sites, visceral metastasis, Ki‐67, pertuzumab‐based regimen as first‐line of treatment, T‐DM1 as a second‐line treatment (when testing for OS). The covariates with a significant effect on OS and PFS1 in univariate analysis were further tested in multivariate analysis. The variable “ICH subgroup” was included in the multivariate model independently on the results of univariate analysis. Our choice was due to the relevance of this variable to our study purposes. SPSS software (SPSS version 21.0, SPSS Inc., Chicago, IL) was used for all statistical evaluations.

## Statement on Preprint

This article has not been posted on a noncommercial preprint server.

## Results

From 2003 through 2017, 738 HER2‐positive metastatic BC patients were retrospectively identified at 45 Italian Cancer Centers. Recruited patients had received at least one cycle of pertuzumab‐based regimen and/or T‐DM1. Main patient and tumor characteristics are listed in Table 1. The median age was 54 years, and 63.4% of these patients had an ECOG PS of 0. Three‐hundred‐nineteen (43.2%) patients had a TP breast cancer. One‐hundred‐sixty (21.7%) had a tumor expressing only one HR, and the remaining 259 patients (35.1%) had HRs negative tumors. The majority of the patients had visceral metastases (68.7%), bone‐exclusive disease was recorded in 51 patients (6.9%), and 239 patients (32.4%) had multiple metastatic sites. As shown in Table 2, after analyzing the distribution of metastatic sites as a function of the IHC characteristics, we observed a significantly higher rate of bone‐only disease in TP and ER or PgR positive tumors compared to HRs negative tumors (8.8 and 9.2% *vs*. 3.6%, respectively; *p* = 0.02). Brain metastases were more frequently observed in patients with ER or PgR‐positive BCs and in HRs negative tumors than in patients with a TP subtype, even though this difference did not reach statistical significance (29.6 and 27.8%, respectively, *vs*. 20.8%; *p* = 0.06). Conversely, no differences were highlighted in terms of visceral metastases distribution by HR status (*p* = 0.94).

**Table 1:** Main baseline characteristics of the study population (*n* = 738)

Characteristics	Patients, *n* (%)
Age at diagnosis of metastatic disease	
Median (range)	54 (26–87)
Histology	
Ductal	634 (85.9)
Lobular	32 (4.3)
Other	72 (9.8)
Metastatic at diagnosis	
Yes	247 (33.5)
No	491 (66.5)
ECOG performance status at diagnosis of metastatic disease	
0	468 (63.4)
1	155 (21)
≥2	11 (1.5)
Unknown	104 (14.1)
HER2‐positive at initial diagnosis	
Yes	627 (85)
No	111 (15)
Immunohistochemical subtype at diagnosis of metastatic disease	
Triple‐positive	319 (43.2)
ER‐ or PgR‐positive	160 (21.7)
HRs‐negative	259 (35.1)
Neoadjuvant chemotherapy	
Yes	441 (59.8)
No	297 (40.2)
Neoadjuvant trastuzumab	
Yes	261 (35.4)
No	477 (64.6)
Neoadjuvant hormonal treatment	
Yes	298 (40.4)
No	193 (26.2)
Pertuzumab‐based treatment in first‐line	
Yes	371 (50.3)
No	367 (49.7)
T‐DM1 administered as second‐line	
Yes	371 (50.3)
No	367 (49.7)
Metastatic sites	
Bone only	51 (6.9)
Visceral	507 (68.7)
Brain	185 (25.1)
Number of metastatic sites	
1	499 (67.6)
2	136 (18.4)
≥3	103 (14.0)

Abbreviations: ER, estrogen receptor; PgR, progesterone receptor.

**Table 2:** Sites of metastases sites according to molecular subtype

Molecular subtype	Bone only	Visceral	Brain
Triple‐positive	28 (8.8%)	221 (69.5%)	66 (20.8%)
ER‐ or PgR‐positive	13 (9.2%)	97 (68.2%)	42 (29.6%)
HRs‐negative	10 (3.6%)	189 (68.2%)	77 (27.8%)
*p*	0.02	0.94	0.06

Abbreviations: ER, estrogen receptor; PgR, progesterone receptor.

Overall, our study cohort included 247 patients (34.3%) with *de novo* metastatic disease, 206 patients (28.6%) with metastasis occurrence within 3 years from diagnosis, and 268 patients (37.2%) with metastatic spread after at least 3 years from initial BC diagnosis (Supporting Information Table S1). When analyzing our results by IHC subtype, we observed that in the TP subgroup almost half of the patients experienced metastasis after the first 3 years (47%), while only 18.3% of patients experimented relapse within this same time‐window, and in 34.1% of patients the disease was metastatic at the onset. Conversely, in patients with HRs negative tumors, we found a higher percentage of early relapses (40.8%) or *de novo* metastatic disease (36.7%), compared to patients who developed metastases after the first 3 years (22.4%). Patients with ER or PgR positive tumors showed an intermediate behavior with respect to the previous two subsets. Their percentage of *de novo* metastatic disease was 28.7%, early relapses (within 3 years) were encountered for 27.3% of patients and the remaining 43.8% had a metastatic recurrence beyond the 3‐year period. When we compared the clinical behavior between these three subsets, namely TP, only one HR‐positive and HRs negative patients, no differences were found regarding the percentage of patients with metastatic disease at the diagnosis (*p* = 0.27), while the tendency of TP and one HR‐positive BC patients to develop less frequently early relapses with respect to HRs negative patients was statistically significant (*p* = 0.001; Supporting Information Table S1).

**Figure 1:**
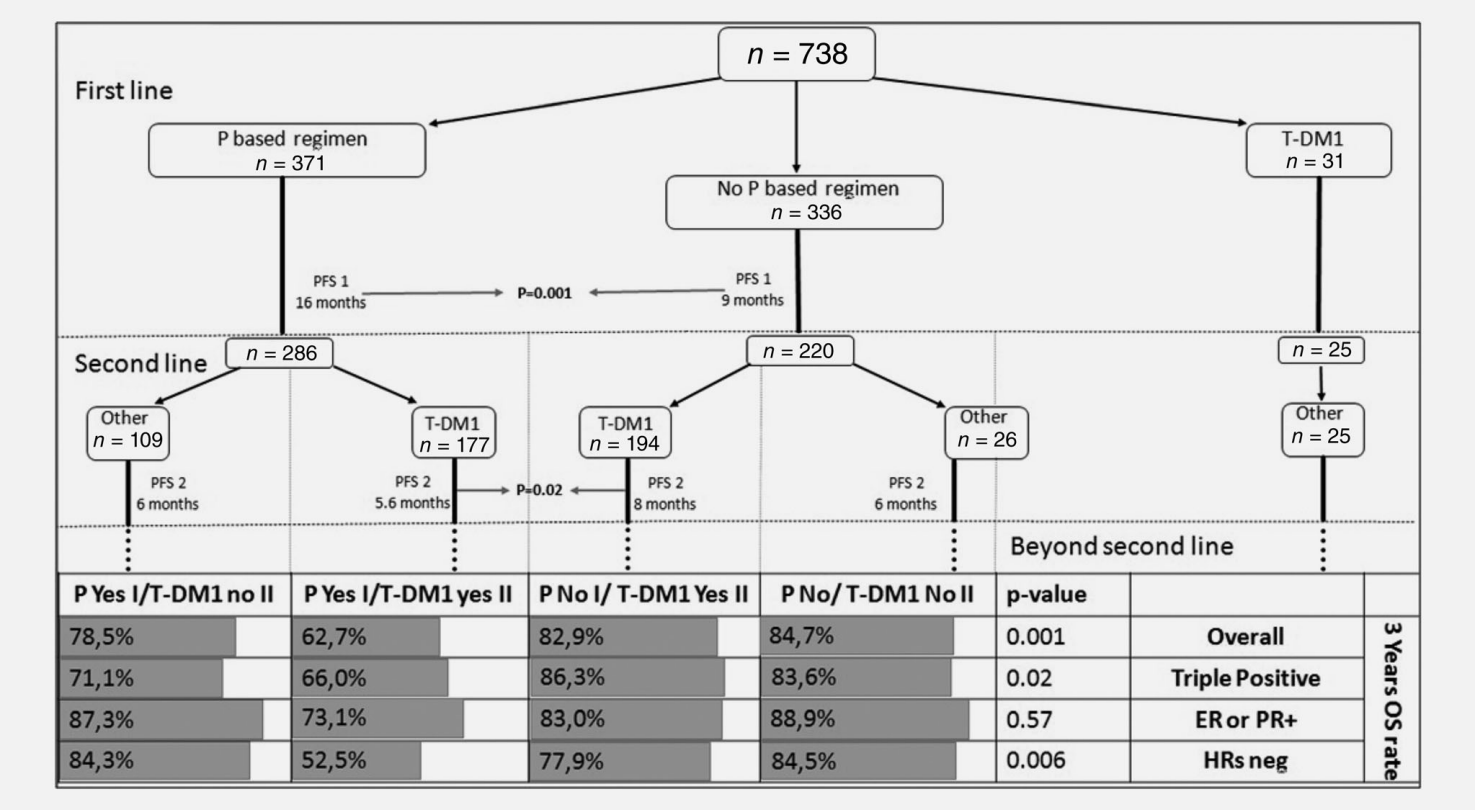
First line and second line of patients divided according to if they received pertuzumab or not in first‐line and T‐DM1 or not in the second‐line. Correlation between sequences of treatment and 3 years OS rate of patients, divided according to immunohistochemistry subtype. Abbreviations: ER, estrogen receptor; HRS, hormone receptors; OS, overall survival; P, pertuzumab; PFS, progression‐free survival; PR, progesterone receptor; P based regimen, pertuzumab‐based regimen; P Yes I, received pertuzumab in first‐line; T‐DM1 Yes II, received T‐DM1 in second‐line; P No I, did not a pertuzumab‐based regimen in first‐line; T‐DM1 No II, did not receive T‐DM1 in second‐line.

Among the 738 patients included in our cohort, 371 (50.3%) received a pertuzumab–trastuzumab–taxane regimen, which was always administered in the first‐line setting. Conversely, a total of 517 (70.1%) patients received T‐DM1, delivered as any line of treatment. In more detail, the number of patients who received T‐DM1 as a first‐, second‐ and third‐line was, respectively, 31 (4.2%), 371 (50.3%) and 96 (13.0%). The remaining 19 (2.6%) patients received T‐DM1 beyond the third‐line of treatment. All the patients treated with T‐DM1 in second‐line or beyond had previously received a pertuzumab‐/trastuzumab‐based or trastuzumab‐based treatment as first‐line. The small cohort of patients (31) who received T‐DM1 as first‐line regimen had experienced a recurrence while on or within 6 months from trastuzumab‐based adjuvant treatment. Overall, a total of 531 (72.0%) patients received a second‐line treatment, including or not T‐DM1. Among the 371 patients who received T‐DM1 as a second‐line of treatment, 177 had been previously treated with a pertuzumab‐/trastuzumab‐based regimen as first‐line, while 194 had been previously treated with a trastuzumab‐based first‐line regimen. Among the 531 patients having received second‐line treatment, 160 received treatment different from T‐DM1 (i.e., lapatinib/capecitabine, trastuzumab/chemotherapy). In these 160 patients, first‐line treatments were represented by a pertuzumab‐based regimen in 109 of them, T‐DM1 in 25 and other treatments in 26 patients (Fig. 1). Patients with HRs‐positive tumors received maintenance endocrine therapy concomitantly with maintenance pertuzumab/trastuzumab, or maintenance trastuzumab after trastuzumab/chemotherapy regimen, whereas patients treated with T‐DM1, did not receive maintenance endocrine therapy after T‐DM1. Moreover, we must take into account that a portion of HRs positive patients after diagnosis of metastatic disease received endocrine therapy plus trastuzumab as first‐line, delaying chemotherapy/Her2‐block. Overall, the median follow‐up was 32.9 months (95% CI, 3–256).

### First‐line treatment

**Figure 2:**
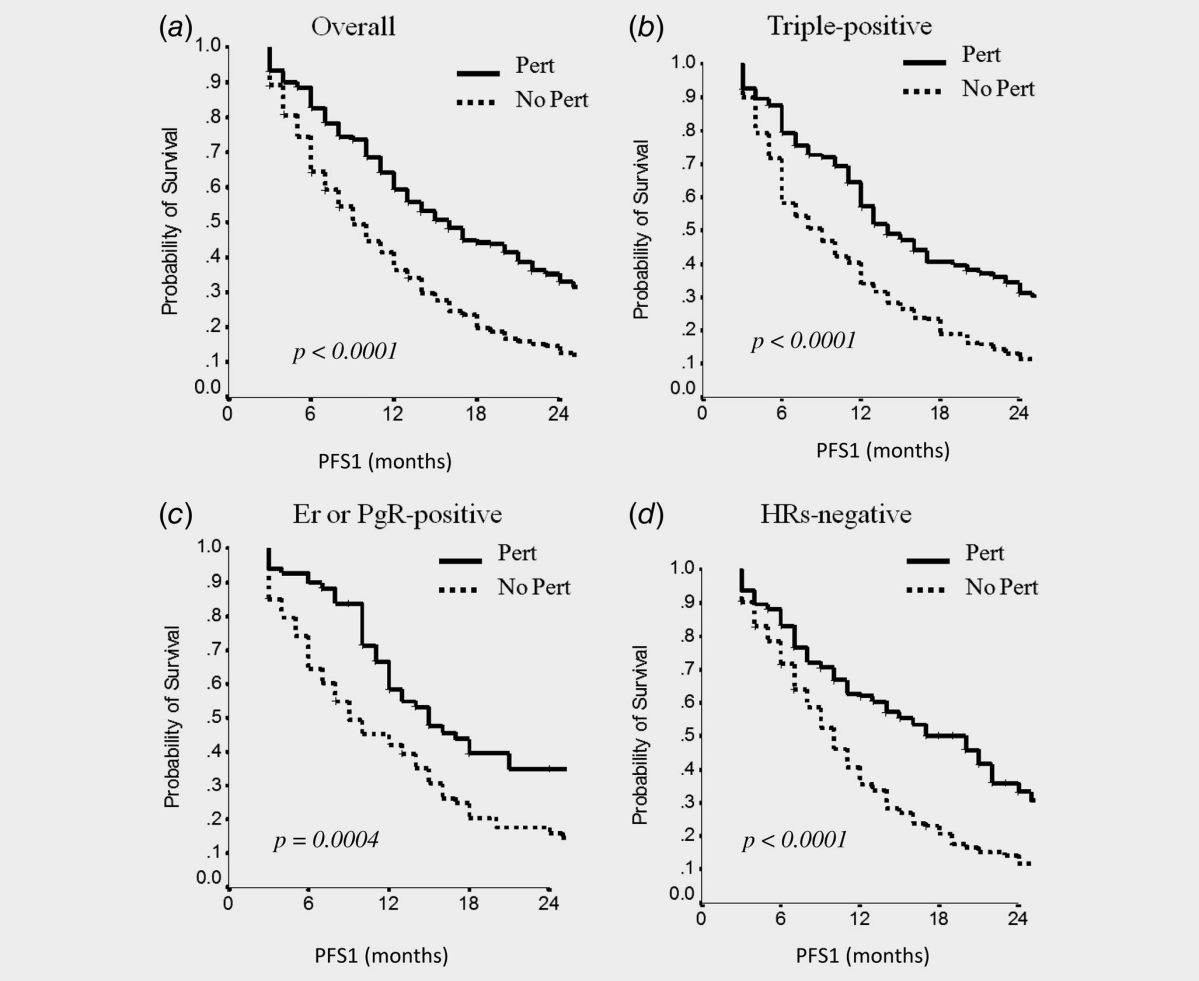
The comparison of progression‐free survival in the first‐line of treatment (PFS1) between patients that received a pertuzumab‐based regimen (Pert) and those who did not (No Pert) in the overall population (*a*), in triple‐positive patients (*b*), in Er‐ or PgR‐positive patients (*c*) and in HRs‐negative patients (*d*). Abbreviations: Er, estrogen receptor; PgR, progesterone receptor; HRs, hormone receptors.

Among the 738 evaluable patients, mPFS at first‐line treatment (mPFS1) was 12 months (95% CI, 11–13), with no significant differences among the IHC subtypes, being 12 months (95% CI, 11–13) in TP subtype, 12 months (95% CI, 10–14) in ER or PgR positive and 12 months (95% CI, 10–14) in HRs negative tumors (*p* = 0.53). Among the 371 patients treated with a first‐line pertuzumab‐/trastuzumab‐based regimen, the overall mPFS was 16 months (95% CI, 13–19), with a significant improvement with respect to patients who did not receive pertuzumab‐based but had received trastuzumab‐based treatments, showing a mPFS of 9 months (95% CI, 8–10; *p* = 0.0001). The advantage in mPFS1 for patients that received pertuzumab is also shown by the PFS1 survival curve in Figure 2 *a*. The mPFS1 benefit related to pertuzumab was observed in all the IHC subtypes at a statistically significant level, as it can be observed in Figures 2 *b*–2 *d*, respectively for TP, ER or PgR positive, and HRs negative subgroups (see also Table 3). In the multivariate analysis (Supporting Information Table S2), the only two variables related to a worse PFS1 were: not having received a pertuzumab‐based regimen as a first‐line treatment (HR 1.7; 95% CI 1.4–2; *p* < 0.0001) and having *de novo* metastatic disease (HR 1.2; 95% CI 1.0–1.4; *p* = 0.042). The multivariate analysis showed no significant effect of the ICH subgroup categories on PFS1.

**Table 3:** Progression‐free survival at first‐line treatment (PFS1) overall and according to molecular subtype and type of treatment received

Molecular subtype	Pertuzumab as first‐line	Median PFS (months, CI)	*p*
Overall	Yes	16 (13–19)	<0.0001
	No	9 (8–10)	
Triple‐positive	Yes	15 (12–18)	<0.0001
	No	8 (6–10)	
ER‐ or PgR‐positive	Yes	15 (10–20)	0.0004
	No	9 (7–11)	
HRs‐negative	Yes	20 (16–24)	<0.0001
	No	9 (8–10)	

Abbreviations: ER, estrogen receptor; PgR, progesterone receptor.

### Second‐line treatment

Among the 531 patients evaluable for second‐line treatment outcome, the median PFS (mPFS2) was 7 months (95% CI, 6–8), with no differences by IHC subtype, being mPFS2 6 months (95% CI, 5–7) in TP, 7 months (95% CI, 5–9) in ER or PgR positive and 8 months (95% CI, 6–10) in HRs negative tumors (*p* = 0.44). Overall, treatment with second‐line T‐DM1 improved PFS, being mPFS2 7 months (95% CI, 5–9) in 371 patients treated with second‐line T‐DM1 and 6 months (95% CI, 5–7) in 160 patients who received as second‐line other HER‐2 based treatments (*p* = 0.003; Tables 1 and 4). However, T‐DM1 advantage in mPFS disappeared when we analyzed the subset of TP patients, which showed a mPFS2 of 6 months (95% CI, 5–7), both with second‐line T‐DM1 and other second‐line treatments (*p* = 0.17). In ER or PgR positive BC patients, mPFS2 was 7 months (95% CI, 4–10) if treated with second‐line T‐DM1, while it was 5 months (95% CI, 3–7) if treated with another regimen (*p* = 0.05). In HRs negative patients, second‐line T‐DM1, compared to other treatments, improved mPFS2, being 10 months (95% CI, 8–12), compared to 7 months of other treatments (95% CI, 6–8), respectively (*p* = 0.04; Table 4). We also addressed the effect of the regimen sequence between first‐line and second‐line treatments on PFS2 (see Fig. 1 for more details), particularly with respect to having received or not a pertuzumab‐based regimen in first‐line, and having received or not T‐DM1 in second‐line. We focused especially on the patients who received T‐DM1 in second‐line, who represent also the major part of subjects who received a second‐line treatment (371 patients, 69.9% of patients who received a second‐line of treatment). Among them, 177 patients who received pertuzumab‐based first‐line had a mPFS2 with T‐DM1 of 5.6 months (95% CI, 4.5–6.6), while the 194 patients who did not receive pertuzumab, but were treated with a trastuzumab‐based first‐line, had a mPFS2 with T‐DM1 of 8 months (95% CI, 6.6–9.6). This difference was statistically significant in the overall population (*p* = 0.02). The longer mPFS2 in patients who received T‐DM1 after a trastuzumab‐based first‐line compared to those who received it after a pertuzumab‐based first‐line regimen was maintained in all the three IHC subgroups (TP, ER or PgR+, HRs−), without significant differences among these three groups (*p* = 0.44). Concerning the160 patients who did not receive T‐DM1 in second‐line, 109 had received pertuzumab in first‐line and showed a mPFS2 of 6 months (95% CI, 4.2–6.8), while the 26 women who were not pretreated with pertuzumab in the first‐line had a mPFS2 of 6 months (95% CI, 4.2–7.8), with no difference between the two groups overall and by ICH subtype. However, our study may be underpowered for an adequate comparison between these latter patients’ subsets. Among the 31 patient having received T‐DM1 as first‐line, 25 received a second‐line. Unfortunately, data on mPFS2 are unavailable.

**Table 4:** Progression‐free survival at second‐line treatment (PFS2) overall and according to molecular subtype and type of treatment received

Molecular subtype	T‐DM1 as second‐line	Median PFS (months, CI)	*p*
Overall	Yes	7 (5–9)	0.003
	No	6 (5–7)	
Triple‐positive	Yes	6 (5–7)	0.17
	No	6 (5–7)	
ER‐ or PgR‐positive	Yes	7 (4–10)	0.05
	No	5 (3–7)	
HRs‐negative	Yes	10 (8–12)	0.04
	No	7 (6–8)	

Abbreviations: ER, estrogen receptor; PgR, progesterone receptor.

### Overall survival

**Figure 3:**
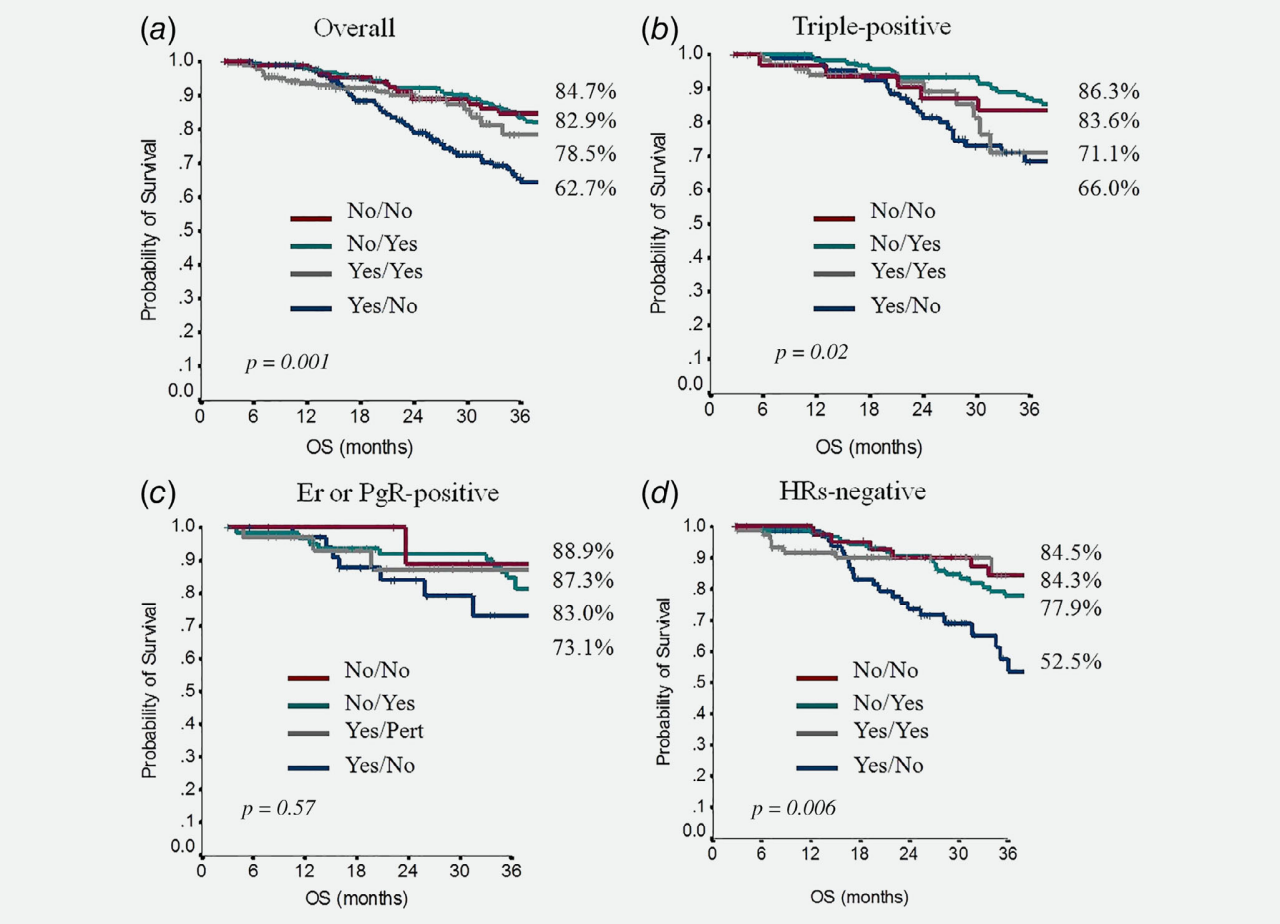
The comparison of overall survival (OS) between patients that did not receive a pertuzumab‐based regimen in first‐line and did not receive T‐DM1 as a second‐line of treatment (No/No, red color), patients that did not receive a pertuzumab‐based regimen in first‐line and received T‐DM1 as a second‐line of treatment (No/Yes, green color), patients that received a pertuzumab‐based regimen in first‐line and T‐DM1 as a second‐line of treatment (Yes/Yes, gray color) and those who received a pertuzumab‐based regimen in first‐line and did not receive T‐DM1 as a second‐line of treatment (Yes/No, blue color). The comparison was done in the overall population (*a*), in triple‐positive patients (*b*), in Er o PgR positive patients (*c*) and in HRs negative patients (*d*). The adjacent percentages to each survival curve refer to the 3‐year survival rate.

Overall, in the 738 patients who contributed data to our analysis, mOS was 74 months (95% CI, 62–87). No differences in mOS emerged when we analyzed our population by IHC subtype, being 73 months (95% CI, 62–87) in TP patients, 78 months (95% CI, 57–98) in ER or PgR positive patients and 76 months (95% CI, 56–95) in HRs negative patients (*p* = 0.61). Furthermore, we analyzed data regarding the rates of OS at 2 and 3 years according to the IHC subtype and treatment received for metastatic disease in first and/or second‐line (Table 5). In the overall population, the administration of pertuzumab‐based regimens as first‐line followed by T‐DM1 in second‐line was associated with significantly lower rates of 2‐ and 3‐year survival with respect to the other possible sequences, as it is clearly shown by the relative survival curves in Figure 3 *a* (*p* = 0.001). The statistical analysis by IHC subtypes showed that this lower OS rate at both 2 and 3 years was statistically significant in the TP and HRs negative subsets (*p* = 0.02 and 0.006, respectively), while no significant differences in OS emerged when we considered the ER or PgR positive patient subgroup (*p* = 0.57). We represented these findings also by calculating the associated comparison of survival curves for TP, ER or PgR positive and HRs negative subgroups, respectively, reported in Figures 3 *b*–3 *d*. In the multivariate analysis (Supporting Information Table S2), the only three variables related to a worse OS were: not having received a pertuzumab‐based regimen as a first‐line treatment (HR 2.0; 95% CI 1.4–2.9; *p* < 0.0001), not having received T‐DM1 in second‐line (HR 1.5; 95% CI 1.1–2.0; *p* = 0.008) and a baseline Ki 67 > 20% (HR 1.59; 95% CI 1.1–2.4; *p* = 0.021). Moreover, multivariate analysis showed no significant effect of the ICH subgroup categories on OS. Thus, the distinctive effect of the HRs expression patterns on OS observed in the nonparametric test emerges only if treatment sequences are considered separately. This confirms the hypothesis of HRs expression relevance when considering the treatment choice.

**Table 5:** Overall survival at 2 and 3 years according to the type of treatment received in first and second‐line and molecular subtype (Tarone Ware test)

	Pertuzumab in first‐line	T‐DM1 in second‐line	2 year OS (%)	3 year OS (%)	*p*
Overall	No	No	88.8	84.7	0.001
	Yes	No	89.1	78.5	
	No	Yes	92.1	82.9	
	Yes	Yes	78	62.7	
Triple‐positive	No	No	87.1	83.6	0.02
	Yes	No	89.1	71.1	
	No	Yes	93.4	86.3	
	Yes	Yes	80.2	66.0	
ER‐ or PgR‐positive	No	No	88.9	88.9	0.57
	Yes	No	87.3	87.3	
	No	Yes	91.8	83.0	
	Yes	Yes	83.9	73.1	
HRs‐negative	No	No	89.9	84.5	0.006
	Yes	No	89.9	84.3	
	No	Yes	90.7	77.9	
	Yes	Yes	72.3	52.5	

Abbreviations: ER, estrogen receptor; PgR, progesterone receptor.

## Discussion

We conducted an observational study of 738 HER2‐positive metastatic BC patients who were treated at 45 Italian Cancer Centers in the time frame between May 2003 and November 2017. All of them had received at least one cycle of pertuzumab‐based treatment in first‐line and/or at least one T‐DM1 cycle in the second or following lines of therapy. Data analysis was performed to assess if the expression of HRs was related to significant differences in terms of key patient‐and/or (other than HR) disease‐related features and/or treatment outcomes.

Within our study population, we observed some differences in disease features according to HRs expression. In more detail, TP patients had a significant higher prevalence of bone‐only disease and a reduced occurrence of brain metastases (*p* = 0.02 and 0.06, respectively). Moreover, in the TP and in patients with tumors with only one HR‐positive tumor, there was also a greater proportion of patients who had a late development of metastases, that is, after the first 3 years from the initial diagnosis, with respect to the HRs negative subgroup (*p* ≤ 0.0001).

When we analyzed the overall benefit from the treatment received for metastatic disease, mPFS1 seemed not to be affected by HRs expression (*p* = 0.53). However, as shown in Table 3, in patients treated with pertuzumab–trastuzumab–taxane regimen, mPFS1 was significantly higher, being 16 months (95% CI, 13–19), compared to 9 months of other treatments (*p* < 0.0001). Interestingly, this improvement in terms of mPFS1 was recorded across all the three IHC subtypes analyzed, suggesting a benefit of pertuzumab‐based treatment independently on HRs expression (Table 3, Fig. 2). This was confirmed by the multivariate analysis which also showed that HRs expression pattern did not have a confounding effect on this outcome.

Among the 531 patients evaluable for the second‐line treatment outcomes, overall, mPFS2 to T‐DM1 treatment was 7 months, in comparison with 6 months for the other second‐line treatments (*p* = 0.003). The improvement related to second‐line T‐DM1 did not emerge when considering only TP patients, which had a mPFS2 of 6 months for both T‐DM1 and for other treatments (*p* = 0.17, Table 4). Conversely, in HRs negative tumors, the benefit of second‐line T‐DM1 was clearly evident, with 10 months of mPFS2 *vs*. 7 months of other treatments (*p* = 0.04). These findings were based on a calculation performed on all patients who received a second‐line, without making any distinction between those who had received a pertuzumab‐based regimen in the first‐line and those who had not. The intriguing result that shows a relatively higher mPFS2 advantage in HER2+ HRs negative BC patients supports the hypothesis according to which HRs negative tumors might have a higher sensitivity to chemotherapy and HER2‐blocking agents.^12^ Whereas, the absence of a mPFS2 advantage associated with T‐DM1 compared to other treatments in TP patients, besides the possible lower sensitivity of this subgroup to HER2‐blockers, may be also at least partly attributed to the fact that patients with HRs positive tumors received endocrine maintenance therapy after trastuzumab or trastuzumab‐/pertuzumab‐based therapy. This was not an option for patients who received T‐DM1. The additive effect of these two factors may represent a plausible explanation for the lack of an evident advantage by T‐DM1 in TP BC patients with respect to other therapeutic options. Moreover, results showed that among the371 patients who received T‐DM1 as second‐line, those receiving it after first‐line pertuzumab (N:177)had a mPFS2 of 5.6 months, which was significantly lower than the 8 months of mPFS2 for those patients who received T‐DM1 in second‐line after a first‐line regimen not containing pertuzumab (*n* = 194; *p* = 0.02). This evidence supports a lower efficacy of this agent when delivered immediately after pertuzumab, independently on the ICH subtype. This finding suggests a possible cross‐resistance mechanism between the pertuzumab–trastuzumab double‐block and T‐DM1, which is stronger than a possible similar cross‐resistance mechanism existing between trastuzumab and T‐DM1.

No differences in OS emerged when we analyzed our entire population by IHC subtype. We also analyzed the clinical outcomes of our study population on the basis of the treatment‐sequences received for metastatic disease. Overall, the administration of pertuzumab‐based regimens as first‐line and subsequently T‐DM1 as second‐line was significantly associated with a lower rate of 2‐ and 3‐year survival (*p* = 0.001, Table 5). This result is congruent with what we observed when analyzing mPFS2. However, differently than mPFS2 (where this lower benefit was present in all the IHC subtypes), when analyzing OS, the worse outcome of the sequence pertuzumab ➔T‐DM1 in first and second‐line did not emerge for all the three IHC subgroups. In fact, these unfavorable OS rates for sequential pertuzumab ➔T‐DM1 treatments were statistically significant both in TP and in HRs negative subsets (*p* = 0.02 and 0.006, respectively), but not in patients with ER or PgR positive tumors (*p* = 0.57). This finding could be due to some underlying biological mechanism that needs interpretation, although selection bias and confounding factors cannot be excluded since the effect of the IHC subtype on OS was not confirmed in multivariate analysis.

Among HER2‐positive tumors, the expression pattern of HRs apparently defines distinct subtypes. Specifically, the TP subtype could be considered as the subset which most closely resembles the luminal‐like tumors, in comparison to HER2 overexpressing tumors that express only one or none of the HRs.^9^, ^10^ This hypothesis is supported in our case series by the fact that TP patients had a significant higher prevalence of bone‐only disease, a lower prevalence of brain metastases, and showed a greater proportion of “late” metastases, reinforcing the data already published in this regard.^13^ Moreover, TP patients showed distinctive characteristics also regarding treatment outcomes, since the subgroup showed no PFS benefit and lower OS rates, together with HRs negative subset, when receiving T‐DM1 in second‐line. Therefore, the contemporary expression of HER2 and both HRs may represent a relevant element to be considered when choosing the treatment strategy.

To the best of our knowledge, this is the first study to evaluate the efficacy of new anti‐HER2 therapeutic strategies in relation to HRs expression in metastatic BC patients, while making a clear distinction between patients with HER2‐positive BC that express both HRs, only one of them, or no HRs at all. A differential sensitivity to combined HER2‐blocking agents and chemotherapy according to HR status was consistently reported both in the early and in the advanced setting, giving some clues on the possible role of maintenance endocrine therapy.^14^, ^15^, ^16^, ^17^, ^18^, ^19^, ^20^ A retrospective study performed in HER2‐positive metastatic BC patients suggested that an expression of ER in ≥30% of tumor cells was predictive of reduced response to chemotherapy plus trastuzumab, but at the same time it indicated patients that could get a benefit from a maintenance endocrine treatment added to trastuzumab administration after the induction with chemotherapy and trastuzumab.^20^ Further data confirm the important role of maintenance endocrine treatment in HER2+ mBC patients also when treated with the pertuzumab–trastuzumab double block.

Since 2012, a paradigm shift was observed in the management of HER2‐positive metastatic BC after the results from the CLEOPATRA,^21^ the EMILIA^7^ and the TH3RESA trials.^11^ The first study demonstrated a significant increase in both PFS and OS when adding pertuzumab to trastuzumab plus docetaxel. This benefit was less evident in HR‐positive BC patients. However, these patients did not receive maintenance hormonal therapy. Recently, our group carried out a retrospective observational study in 264 HER2‐positive metastatic BC patients treated with a pertuzumab‐based regimen as a first‐line. Results were consistent with the findings from the CLEOPATRA trial. Differently from the pivotal trial, in our patient population maintenance endocrine therapy was added to pertuzumab–trastuzumab maintenance in 103 patients, who had the most favorable clinical outcomes in terms of PFS and OS, suggesting that the double‐maintenance therapy (HER2 blockade and endocrine treatment) could have a relevant positive clinical impact in patients with HER2‐positive/HR‐positive BC.^22^ In the present analysis, patients treated with pertuzumab‐/trastuzumab‐based regimens experienced a mPFS1 that was comparable to that of the pivotal trial, being 16 months, *vs*. 9 months in those patients who received other first‐line treatments (*p* < 0.0001). A clear advantage from pertuzumab‐/trastuzumab‐based regimens was evident in all analyzed IHC subtypes, including TP and one HR‐positive subgroups, who, in our study cohort, received also endocrine maintenance treatment, which may theoretically “regain” the “PFS loss” in the HRs‐positive patients, presumably less responsive to HER2‐blocking agents. Besides the proven impact of endocrine treatment received in the metastatic setting, we performed further analysis to evaluate whether endocrine treatment received in the neoadjuvant or adjuvant setting may have had an impact on mPFS1 and mPFS2. As we show in Supporting Information Table S3, among the 491 patients who were initially diagnosed with an early or locally advanced disease, 298 received endocrine treatment as neoadjuvant or adjuvant therapy, while 193 did not. Overall, the patients who had received an endocrine therapy in the early setting had a mPFS1 of 12 months *vs*. 11 months of patients who did not. This difference was not significant (*p* = 0.14). Neither significant differences were found when comparing the mPFS2 of these same two groups (*p* = 0.54). When this same comparison was performed in patients stratified by IHC subgroups, in TP patients, who had received endocrine therapy in the early setting, we verified some impact on the mPFS1 outcome. In fact, mPFS1 for TP patients who had received neoadjuvant/adjuvant endocrine therapy was 11 months compared to 16 months of patients who did not. This difference was at the limit of statistical significance (*p* = 0.05). We may thus hypothesize that the worse outcome in terms of PFS1 for TP patients having received endocrine therapy in the early setting may be related to the fact that maintenance endocrine treatment for this patients yielded less PFS1 advantage due to the onset of resistance to antihormonal agents. At the same time, this result confirms, at least indirectly, the positive impact of endocrine maintenance therapy in TP patients for PFS1. In this same analysis, no relevant impact on mPFS2 was found for endocrine treatment in the early setting of TP patients (*p* = 0.26). Moreover, the TP‐positive subgroup, as defined based on IHC, was the only one to show some impact of endocrine treatment in early setting on outcomes verified in the metastatic setting. In fact, for both the remaining subgroups, ER‐ or PR‐positive patients and HRs‐negative patients, administration of endocrine treatment in the early setting did not impact either mPFS1 (respective *p* values were 0.48 and 0.86) or mPFS2 (respective *p* values were 0.92 and 0.24). It is noteworthy that the 41 patients in the HRs IHC subgroup, who had received endocrine treatment in the early setting, had initially an HRs‐positive disease. Overall, this further analysis confirms the distinct nature of TP disease and the particular importance of antihormonal treatment in this subgroup.

The EMILIA trial compared T‐DM1 to lapatinib and capecitabine for treatment of advanced HER2‐positive BC in second‐line or beyond.^7^ The experimental arm had superior PFS and OS. Subgroup analysis failed to show a differential impact of the treatment based on HRs status. Our group carried out a retrospective, observational analysis of T‐DM1 clinical activity in 250 HER2‐positive metastatic BC patients.^23^ Overall, our results were compared to those from randomized trials and, similarly to what was found in those studies, no differences in clinical outcomes emerged when we analyzed our population by HRs status (*p* = 0.29). However, when analyzed in relation to pertuzumab‐pretreatment, patients who received second‐line T‐DM1 had mPFS2 and mOS of 3 and 12 months, respectively (*p* = 0.0001) when pertuzumab‐pretreated, and 8 and 26 months when pertuzumab‐naïve (*p* = 0.06). Conversely, in third‐line and beyond, mPFS and mOS to T‐DM1 were 16 and 18 months in pertuzumab‐pretreated (*p* = 0.05), and 6 and 17 months in pertuzumab‐naïve patients (*p* = 0.30). The results of our study regarding the PFS of the second‐line of treatment and OS are consistent with those from the present study analyzing a larger population, when pertuzumab pretreatment is considered. In fact, in the current analysis, patients pretreated with pertuzumab who received T‐DM1 in second‐line had a PFS advantage with respect to those not receiving second‐line T‐DM1 (*p* = 0.03). Moreover, in the cohort of the present study, receiving T‐DM1 in second‐line, immediately after pertuzumab, resulted detrimental in terms of OS with respect to receiving it after a trastuzumab‐based first‐line. As we mentioned before, this might be related to a possible transient cross‐resistance between the two agents, although the outcome could be explained also by a selection bias, considering that patients who received T‐DM1 as third or more advanced lines presumably had a more indolent disease which allowed several lines of treatments, compared to patients treated with T‐DM1 as second‐line. In the current study, a further analysis to explore the OS rates in the different IHC subgroups showed that the aforementioned difference in OS rates according to sequential pertuzumab and T‐DM1 treatment was statistically significant in the TP and HRs negative subsets (*p* = 0.006). Conversely, it was not recorded in patients with ER‐ or PgR‐positive tumors (*p* = 0.57). Specifically, TP BC could have more potential to develop chemotherapy resistance related to the activation of the ER pathway.^24^ This mechanism could be less evident in HER2‐positive tumors that express low levels of HRs or only one of them. At the same time, our results in HRs negative tumors might be related to an intrinsic aggressiveness of the disease in this subset.^25^

Overall, our results are consistent with some previously emerged evidences on the reduced activity of T‐DM1 when given immediately after pertuzumab‐based regimens. A retrospective study investigating the efficacy of T‐DM1 after pertuzumab‐based combination therapy showed shorter median duration of T‐DM1 therapy (4.0 months) in patients who had received prior pertuzumab‐based regimens. When discussing this latter evidence, the authors ascribed it at least partially to the retrospective nature of the research and the relatively high percentage of *de novo* stage IV patients (44%).^26^ However, the present study included only 33.5% of *de novo* stage IV patients. Therefore, it is plausible that the lower efficacy of T‐DM1 in patients pretreated with pertuzumab‐/trastuzumab‐based combinations might be the result of other mechanisms.

Evidence of lower efficacy of T‐DM1, when administered immediately after a pertuzumab‐based regimen, was also found in prospective studies. The preliminary results of the PERNETTA study conducted in 210 patients with HER2‐positive metastatic BC treated with a pertuzumab‐containing regimen in first‐line and with T‐DM1 as second‐line, showed that mPFS in second‐line treatment was lower (5.3 months) than that recorded in the pivotal trial, where patients treated with second‐line T‐DM1 were pertuzumab‐naïve.^27^ Furthermore, a recently published prospective study carried out in 42 Japanese HER2‐positive metastatic BC patients has shown that the mPFS after T‐DM1 administration was lower in the group pretreated with pertuzumab‐/trastuzumab‐based regimens compared to the group of patients who received trastuzumab‐based regimens (2.8 months *vs*. 7.8 months, respectively, *p* = 0.0030).^28^

The present study has some limitations. It has a retrospective design, which *per se* represents a source of confounding and bias. Selection bias may be fueled by the intrinsic characteristics of our patients’ disease, which may have affected at some extent treatment outcomes. In more detail, patients who received T‐DM1 as third or more advanced lines might have a more indolent disease which allowed them to receive T‐DM1 after other treatment‐lines. In addition, since we address treatment outcomes by cancer subtype, the lack of centralized evaluation by either IHC or NGS techniques may lessen our confidence in the results observed, although quality controls are in place at the participating centers. In addition, for patients who provided data to this analysis, the follow‐up period was relatively short. Our study also has several strengths. This is the first report to address outcomes of treatment sequences in a quite large cohort of HER2‐positive metastatic BC patients in light of the biological characteristics of the disease, with a specific focus on HRs expression. In addition, our study was conducted in patients treated outside clinical trials. As such, it faithfully reproduces the current clinical practice. Numerous are the evidences related to the biological differences and response to treatment within the subgroup globally defined as HER2‐positive BC. This is the first report that has consistently shown a response to novel anti‐HER2 treatments differing by HRs status. Since HER2‐positive BC evolves under the selective pressure of targeted agents, it is of paramount importance recognizing resistance pathways related to the exposure to novel treatments according to a given sequence. Our results provide a strong rationale to perform studies aiming at a deeper understanding of the intrinsic biology of HER2‐positive disease, in order to optimally combine the available hormonal and anti‐HER2 therapy treatments to overcome both endocrine and anti‐HER2 resistance. The final goal is the achievement of decisions driving to the most effective therapeutic choice at an individual patient level.

In conclusion, breast cancer biology is of uttermost relevance for optimizing treatment and better interpreting the clinical outcomes. The central role of the HRs and the HER2 pathways in sustaining BC development and growth has been clearly established. The expression of both HRs concomitantly with the overexpression of HER2 depicts a TP subtype that may have distinct clinical manifestation and treatment outcomes compared to the other HER2‐positive subtypes. Results from this historical cohort support our hypothesis. In more details, in patients with TP tumors, we actually observed clinical manifestations that more closely resemble those of luminal A patients, with higher rates of bone‐only disease and lower rate of brain metastasis. No differences emerged in terms of first‐line treatment outcomes in TP patients with respect to the other subsets, is the benefit of pertuzumab‐based treatment consistent in all subgroups. However, the PFS gain due to the administration of T‐DM1 as second‐line that was observed in the overall population seems to disappear when analyzing the TP subgroup, giving a hint on a possible additional mechanism of resistance towards HER2‐blockers in this subset of patients. On this basis, our data confirm the need for further exploration of TP BC throughout the conduct of *ad‐hoc* designed, appropriately sized, prospective trials. These latter studies will be aimed to clarify the extent to which treatment outcomes in HER2‐positive metastatic BC treated with sequences including hormonal therapy and novel anti‐HER2 agents may differ depending on the coexpression of both the HRs *vs*. only one of the two. This research will provide data on “canonical” treatment outcomes, which may be more appropriately interpreted in light of the results from the annexed tasks and experimental designs focused on the underlying mechanisms regulating the development of resistance across the different subtypes of HER2‐positive metastatic BC patients. A possible equalizer for outcome differences in TP patients maybe a wider use of concomitant antihormonal therapy when possible.

## Supporting information


**Table S1** Elapsed time between the diagnosis of cancer and the development of metastasis

**Table S2** PFS 1 and OS multivariate analysis

**Table S3** Correlation between endocrine treatment in the neoadjuvant setting and mPFS (first line and second line of treatment) overall and by subgroups defined by immunohistochemistry, that is, triple positive, ER‐ or PR‐positive and HRs negative subgroup

## Data Availability

Raw data were generated at Regina Elena National Cancer Institute—IFO and collected from other 44 Italian Cancer Centers. Derived data supporting the findings of our study are available from the corresponding author (E.K.) on request.
