# Supplementary material for: Distinct HR expression patterns significantly affect the clinical behavior of metastatic HER2+ breast cancer and degree of benefit from novel anti‐HER2 agents in the real world setting
Source: Int J Cancer. 2019 Aug 7;146(7):1917–29. doi: 10.1002/ijc.32583 (PMC7027476; doi:10.1002/ijc.32583)
Supplement: Supplementary file 1 — Table S1 Elapsed time between the diagnosis of cancer and the development of metastasis [file IJC-146-1917-s001.docx]

Supplementary Table 1. Elapsed time between the diagnosis of cancer and the development of metastasis

| *Molecular Subtypes* | Metastases at diagnosis, N (%) | Metastases< 3 years from diagnosis, N (%) | Metastases≥ 3 years from diagnosis, N (%) |
| --- | --- | --- | --- |
| Overall | 247 (34.3% of total pts) | 206 (28.6% of total pts) | 268 (37.2% of total pts) |
| Triple Positive | 107 (34.1%) | 57 (18.3%) | 146 (47%) |
| ER or PgR Positive | 40 (28.7%) | 38 (27.3%) | 61 (43.8%) |
| HRs negative | 100 (36.7%) | 111 (40.8%) | 61 (22.4%) |
| *p-value* | *0.27* | *0.001* | *0.001* |

Abbreviations: ER, estrogen receptor; PgR, progesterone receptor; pts, patients
