# Supplementary material for: Distinct HR expression patterns significantly affect the clinical behavior of metastatic HER2+ breast cancer and degree of benefit from novel anti‐HER2 agents in the real world setting
Source: Int J Cancer. 2019 Aug 7;146(7):1917–29. doi: 10.1002/ijc.32583 (PMC7027476; doi:10.1002/ijc.32583)
Supplement: Supplementary file 2 — Table S2 PFS 1 and OS multivariate analysis [file IJC-146-1917-s002.docx]

|  | **PFS 1 Multivariate analysis** | | | **OS multivariate analysis** | | |
| --- | --- | --- | --- | --- | --- | --- |
|  | **HR** | **C.I.** | **p-value** | **HR** | **C.I.** | **p-value** |
| **Subtype**  - TP vs other  - ER+ or PR+ vs other  - HRs neg vs other | / | / | / | / | / | / |
|  | 1.074 | 0.898-1.284 | 0.436 | 0.961 | 0.721-1.280 | 0.784 |
|  | 0.974 | 0.780-1.227 | 0.849 | 0.908 | 0.631-1.300 | 0.605 |
|  | 0.931 | 0.779-1.114 | 0.436 | 1.041 | 0.781-13.87 | 0.784 |
| **Metastasis at diagnosis (Yes/No)** | 1.205 | 1.007-1.442 | 0.042 | NA | NA | NA |
| **Ki67 (≥ 20%/<20%)** | NA | NA | NA | 1.59 | 1.073-2.357 | 0.21 |
| **First line with Pertuzumab (No/Yes)** | 1.688 | 1.425-1.999 | <0.0001 | 2.037 | 1.414-2.934 | <0.0001 |
| **Second line with T-DM1 (No/Yes)** | NA | NA | NA | 1.486 | 1.108-1.994 | 0.0008 |

**Supplementary Table 2**: PFS 1 and OS multivariate analysis.

C.I.: confidence interval; ER+: estrogen receptor positive; HR: hazard ratio; HRs: hormone receptors OS: overall survival; PFS: progression free survival; PR+: progesteron receptor positive
