# Supplementary material for: Distinct HR expression patterns significantly affect the clinical behavior of metastatic HER2+ breast cancer and degree of benefit from novel anti‐HER2 agents in the real world setting
Source: Int J Cancer. 2019 Aug 7;146(7):1917–29. doi: 10.1002/ijc.32583 (PMC7027476; doi:10.1002/ijc.32583)
Supplement: Supplementary file 3 — Table S3 Correlation between endocrine treatment in the neoadjuvant setting and mPFS (first line and second line of treatment) overall and by subgroups defined by immunohistochemistry, that is, triple positive, ER‐ or PR‐positive and HRs negative subgroup [file IJC-146-1917-s003.docx]

|  | Neoadjuvant/adjuvant  Endocrine therapy | |  |  |
| --- | --- | --- | --- | --- |
|  | **NO** | **YES** | **Value** | **p** |
| **OVERALL** | N: 193 | N: 298 | Nr patients | / |
|  | 12 months  (10-14) | 11 months  (10-12) | mPFS1  (range) | 0.14 |
|  | 7 months  (5-9) | 6.6 months  (6-7) | mPFS2  (range) | 0.54 |
| **TRIPLE POSITIVE** | 34 | 178 | Nr patients | / |
|  | 16 months  (13-19) | 11 months  (10-12) | mPFS1  (range) | 0.05 |
|  | 7 months  (3-11) | 6 months  (5-7) | mPFS2  (range) | 0.26 |
| **ER or PR +** | 23 | 79 (10.8) | N (%) | / |
|  | 14 months  (11-17) | 10 months  (8-12) | mPFS1  (range) | 0.48 |
|  | 19 months  (0-38) | 6 months  (5-8) | mPFS2  (range) | 0.92 |
| **HRs negative** | 136 | 41 | Nr patients | / |
|  | 10 months  (8-12) | 12 months  (8-16) | mPFS1  (range) | 0.86 |
|  | 7 months  (5-9) | 14 months  (8-20) | mPFS2  (range) | 0.24 |

**Supplementary Table 3**: correlation betweenendocrine treatment in the neo/adjuvant setting and mPFS (first line and second line of treatment) overall and by subgroups defined by immunohistochemistry, i.e., triple positive, ER or PR positive, and HRs negative subgroup.

ER: Estrogen Receptor; HRs: hormonal receptors; PFS: progression free survival; PR: Progesteron Receptor
